# FGFR1 is an adverse outcome indicator for luminal A breast cancers

**DOI:** 10.18632/oncotarget.6563

**Published:** 2015-12-11

**Authors:** Yu-Jie Shi, Julia Y.S. Tsang, Yun-Bi Ni, Siu-Ki Chan, Kui-Fat Chan, Gary M. Tse

**Affiliations:** ^1^ Department of Pathology, Henan Province People's Hospital, Zhengzhou, Henan, China; ^2^ Department of Anatomical and Cellular Pathology, Prince of Wales Hospital, The Chinese University of Hong Kong, Shatin, NT, Hong Kong; ^3^ Department of Pathology, Kwong Wah Hospital, Kowloon, Hong Kong; ^4^ Department of Pathology, Tuen Mun Hosiptal, Tuen Mun, Hong Kong

**Keywords:** fibroblast growth factor receptor 1, breast cancer, luminal subtype, immunohistochemistry

## Abstract

Fibroblast growth factor receptor 1 (FGFR1) has been suggested to be the candidate gene for 8p11–12 amplification in breast cancer and its therapeutic/prognostic value is explored. Most previous studies focused on FGFR1 gene amplification, which may not necessarily lead to protein expression. Therefore, analysis of protein level may have more clinical relevance. We evaluated FGFR1 expression in a large cohort of breast cancer by immunohistochemistry, correlated with the tumor clinic-pathologic features, biomarkers expression, and patient's survival. FGFR1 expression was associated mainly with luminal cancers, particularly luminal B subtype (23.5%; *p* < 0.001), and it also showed adverse prognostic impact on luminal A cancers. FGFR1 expression was associated with higher pN (*p* = 0.023), pT (*p* = 0.003) stages, lymphovascular invasion (*p* = 0.010), p-cadherin (*p* = 0.028), synaptophysin (*p* = 0.009) and SOX2 expression (*p* = 0.034) in luminal A cancers. FGFR1 expressing luminal A cancers showed a similar outcome as luminal B cancers. Multivariate Cox regression analysis demonstrated FGFR1 positive luminal A cancers to be an independently poor prognosticator for disease free survival in luminal cancers (hazard ratio = 3.341, *p* = 0.008). Thus FGFR1 could be useful in identifying the aggressive cases amongst heterogeneous luminal A cancers. Given the relevance of FGFR pathway in treatment resistance in luminal cancers, FGFR1 could be an important tumor biomarker and adverse prognostic factor potentially exploitable in the clinical management of luminal cancers.

## INTRODUCTION

Breast carcinogenesis may involve genetic alterations including activation or amplification of oncogenes [1]. Amplification at 8p11–12 is frequent, being reported in approximately 10–15% of breast cancers [2, 3]. Fibroblast growth factor receptor 1 (*FGFR1*) which encodes for a tyrosine kinase receptor in the FGFR family (FGFR1–4), is suggested to be the candidate gene [2, 4].

FGFR1 plays critical functions in the normal mammary physiologic development and tissue homeostasis. It is expressed in the mammary epithelium during ductal morphogenesis. Prenatal deletion of FGFR1 resulted in delayed mammary gland development and a transient reduction in cellular proliferation [5]. In breast cancers, FGFR1 is mainly localized in the cytoplasm and cell membrane. Aberrant FGFR regulation or expression induced mammary tumor cell proliferation, anti-apoptosis, drug resistance, epithelial-to-mesenchymal transition (EMT) and invasion [6, 7]. Recent investigations have highlighted the potential clinical values of FGFR1 as a therapeutic target and prognostic biomarker in breast cancers. FGFR1 amplification might be important in the invasive transition processes [8]. More FGFR1 amplification was observed in invasive breast cancer than the non-invasive ductal carcinoma *in situ* [9]. In addition, FGFR1 amplification was associated with distant metastasis, early relapse and poor survival [3, 9–11], and contributed to poor prognosis in luminal breast cancers by driving anchorage independent proliferation and endocrine therapy resistance [10]. In triple negative breast cancers (TNBC), the role of FGFR1 is less clear. While one study showed no effect of FGFR1 amplification on patient survival [12], another study showed FGFR1 protein expression correlated with decreased OS [13]. Whether this discrepancy was related to analysis at gene or protein levels was uncertain. It was important to note that FGFR1 gene amplification did not necessarily lead to a high protein expression, as low protein expression level in FGFR1 amplified tumors had also been observed [10].

While most previous studies focused on the FGFR1 amplification in clinical breast cancers as a group, FGFR1 protein expression in different molecular breast cancer subtypes and its association with other important biomarkers and prognosis are far from clear. In this study, the expression of FGFR1 in a large cohort of breast cancer was evaluated and correlated with various clinic-pathological features, biomarker expression and outcome, as well as with different breast cancer molecular groupings.

## RESULTS

**Figure 1:**
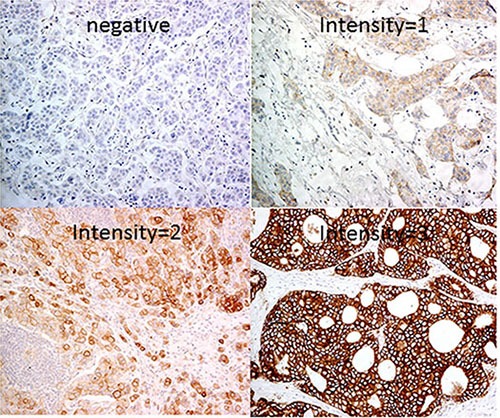
Representative immunohistochemical stainings of FGFR1 (200x)

A total of 1,093 primary invasive breast cancers were included in this cohort. Details of the clinic-pathological features are summarized in Table 1. Overall, 941 cases (86.1%) were negative for FGFR1 and 152 cases (13.9%) were positive. Representative FGFR1 staining is shown in Figure 1.

**Table 1:** Correlations of FGFR1 expression with clinic-pathological features

	FGFR1 Negative (%)	FGFR1 Positive (%)	Total	*p* Value
Grade				0.136
1	144 (90)	16 (10)	160	
2	375 (86.0)	61 (14.0)	436	
3	422 (84.9)	75 (15.1)	497	
FF				0.073
Absence	700 (87.3)	101 (12.6)	801	
Presence	220 (83.0)	45 (17.0)	265	
Total	920 (86.3)	146 (13.7)	1066	
LVI				0.066
Absence	651 (87.6)	92 (12.4)	743	
Presence	244 (83.3)	49 (16.7)	293	
Total	895 (86.4)	141 (13.6)	1036	
pN				**0.042**
0	481 (88.3)	64 (11.7)	545	
1	265 (83.9)	51 (16.1)	316	
2	113 (87.6)	16 (12.4)	129	
3	68 (79.1)	18 (20.9)	86	
Total	927 (86.2)	149 (13.8)	1076	
pT				**0.037**
1	389 (88.2)	52 (11.8)	441	
2	470 (84.5)	86 (15.5)	556	
3	48 (82.8)	10 (17.2)	58	
4	13 (76.5)	4 (23.5)	17	
Total	920 (85.8)	152 (14.2)	1072	
Molecular				**< 0.001**
Lum A	403 (89.6)	47 (10.4)	450	
Lum B	287 (76.5)	88 (23.5)	375	
HER2-OE	106 (95.5)	5 (4.5)	111	
BLBC	60 (88.2)	8 (11.8)	68	
5NP	77 (93.9)	5 (6.1)	82	
Age				0.062
Mean	55 (100.5)	52.3 (95.6)	54.7	
SD	12.9 (100.8)	12 (93.8)	12.8	
Range	22–97	28–91		
Tumor size				**0.017**
Mean	2.63	2.9	2.67	
SD	1.491	1.5	1.5	
Range	0.2–13.9	0.3–9.5		

BOLD: statistically significant

### Correlation with tumor clinic-pathological characteristics, molecular subtypes and biomarkers

FGFR1 expression was found to be associated with high pN (*p* = 0.042), pT (*p* = 0.037) stages and large tumor size (*p* = 0.017), but not with tumor grade, LVI, FF and patients’ age (Table 1).

Among the 1086 invasive cancers with complete data for IHC based molecular classification, 450 (41.4%) were Lum A, 375 (34.5%) were Lum B, 111 (10.2%) were HER2-OE and 150 (13.9%) were TNBC (including 68 cases (6.3%) of BLBC and 82 cases (7.6%) unclassified). The expression rate of FGFR1 was 10.4% in Lum A, 23.5% in Lum B, 4.5% in HER2-OE, and 17.9% in TNBC (11.8% in BLBC and 6.1% in unclassified) cancers. Significant difference in FGFR1 expression was found among different molecular subtypes (*p* < 0.001), with the highest expression rate seen in Lum B cancers (Table 1).

For biomarkers, FGFR1 expression correlated with overall high ER, Ki67, P63, SOX2 and markers of neuroendocrine differentiation (CG and SYN) (*p* ≤ 0.001 for all, except *p* = 0.038 for SOX2). There was no significant correlation with other biomarkers, including PR, EGFR, HER2, c-kit, CK5/6, CK14 and P-cadherin (Table 2).

**Table 2:** Correlations of FGFR1 expression with biomarkers

	FGFR1 Negative (%)	FGFR1 Positive (%)	Total	*p* Value
ER				**< 0.001**
Neg	299 (92.3)	25 (7.7)	324	
Pos	641 (83.4)	128 (16.6)	769	
Total	940 (86.0)	153 (14.0)	1093	
PR				0.969
< 20%	411 (86.0)	67 (14.0)	478	
≥ 20%	524 (85.9)	86 (14.1)	610	
Total	935 (86.0)	153 (14.0)	1088	
HER2				0.674
Neg	760 (85.6)	128 (14.4)	888	
Pos	189 (86.7)	29 (13.3)	218	
Total	949 (85.8)	157 (14.2)	1106	
Ki67				**< 0.001**
< 20%	699 (88.7)	89 (11.3)	788	
≥ 20%	234 (78.5)	64 (21.5)	298	
Total	933 (85.9)	153 (14.1)	1086	
P63				**0.001**
Neg	904 (86.5)	141 (13.5)	1045	
Pos	31 (72.1)	12 (27.9)	43	
Total	935 (85.9)	153 (14.1)	1088	
CK5/6				0.814
Neg	837 (86.0)	136 (14.0)	973	
Pos	98 (85.2)	17 (14.8)	115	
Total	935 (85.9)	153 (14.0)	1088	
CK14				0.118
Neg	881 (86.5)	138 (13.5)	1019	
Pos	55 (79.7)	14 (20.3)	69	
Total	936 (86.0)	152 (14.0)	1088	
P-cadherin				0.733
Neg	713 (86.0)	116 (14.0)	829	
Pos	218 (85.2)	38 (14.8)	256	
Total	931 (85.8)	154 (14.2)	1085	
CG				**0.001**
Neg	902 (86.8)	137 (13.2)	1039	
Pos	30 (66.7)	15 (33.3)	45	
Total	932 (86.0)	152 (14.0)	1084	
SYN				**< 0.001**
Neg	854 (87.0)	127 (13.0)	981	
Pos	81 (75.7)	26 (24.3)	107	
Total	935 (86.0)	153 (14.0)	1088	
Sox2				**0.038**
Neg	343 (84.9)	61 (15.1)	404	
Pos	81 (76.4)	25 (23.6)	106	
Total	424 (83.1)	86 (16.9)	510	

*statistically significant

### FGFR1 expression in luminal subtypes

Given the significant correlation of FGFR1 the luminal subtypes, the relationship of FGFR1 with clinical features was investigated for luminal subtypes separately. FGFR1 was expressed in 134 out of 824 cases (16.3%) and 18 out of 261 cases (6.9%) of luminal and non-luminal cancers respectively.

In Lum cancers, FGFR1 expression was associated with high tumor grade (*p* = 0.005), pN (*p* = 0.004) and pT stages (*p* = 0.001), large tumor size (*p* = 0.001), and the presence of LVI (*p* = 0.031) (Table 3). For biomarkers, FGFR1 expression was positively associated with high Ki67 (*p* < 0.001), p-cadherin (*p* = 0.011), CG (*p* = 0.007), SYN (*p* = 0.001) and SOX2 (*p* = 0.013) but negatively with PR (*p* = 0.003). In addition, it was predominantly expressed in luminal B over luminal A subtype (*p* < 0.001) (Supplementary Table S2).

**Table 3:** Association of FGFR1 expression of clinic-pathological features and biomarker expression according to different luminal subtypes

	Luminal A FGFR1 (%)				Luminal B FGFR1 (%)			
	Negative	Positive	Total	*p*-value	Negative	Positive	Total	*p*-value
Clinic-pathological features								
Grade				0.124				0.784
1	110 (92.4)	9 (7.6)	119		25 (78.1)	7 (21.9)	32	
2	213 (89.5)	25 (10.5)	238		114 (77.0)	34 (23.0)	148	
3	79 (85.9)	13 (14.1)	92		147 (76.2)	46 (23.8)	193	
Total	402 (89.5)	47 (10.5)	449		286 (76.7)	87 (23.3)	373	
FF				0.177				0.376
Absence	229 (90.9)	30 (9.1)	329		209 (78.0)	59 (22.0)	268	
Presence	95 (86.4)	15 (13.9)	110		69 (73.4)	25 (26.6)	94	
Total	394 (89.7)	45 (10.3)	439		278 (76.8)	84 (23.2)	362	
LVI				**0.010**				0.972
Absence	309 (92.0)	27 (8.0)	336		172 (76.4)	53 (23.6)	225	
Presence	78 (83.0)	16 (17.0)	94		95 (76.6)	29 (23.4)	124	
Total	387 (90.0)	43 (10.0)	430		267 (76.5)	82 (23.5)	349	
pN				**0.023**				0.279
0	225 (93.4)	16 (6.6)	241		138 (78.0)	39 (22.0)	177	
1	117 (84.2)	22 (15.8)	139		76 (77.6)	22 (22.4)	98	
2	38 (92.7)	3 (7.3)	41		42 (77.8)	12 (22.2)	54	
3	14 (77.8)	4 (22.2)	19		27 (67.5)	13 (32.5)	40	
Total	394 (89.7)	45 (10.3)	439		283 (76.7)	86 (23.3)	369	
pT				**0.003**				0.330
1	207 (93.2)	15 (6.8)	222		102 (77.9)	29 (22.1)	131	
2	174 (86.1)	28 (13.9)	202		156 (75.7)	50 (24.3)	206	
3	10 (76.9)	3 (23.1)	13		17 (77.3)	5 (22.7)	22	
4	3 (75.0)	1 (25.0)	4		3 (50.0)	3 (50.0)	6	
Total	394 (89.3)	47 (10.7)	441		278 (76.2)	87 (23.8)	365	
Age				0.282				0.720
Mean	56.7	54.5	56.4		52.3	52.0	52.2	
SD	13.1	13.6	13.1		12.2	11.2	12.0	
Range	30–97	28–91			22–85	31–89		
Tumor size				**0.005**				0.311
Mean	2.31	2.91	2.37		2.81	2.94	2.84	
SD	1.16	1.54	1.22		1.78	1.61	1.73	
Range	0.2–9.0	0.3–7.2			0.3–13.9	0.5–9.5		
Biomarker								
ER				0.490				0.137
Neg	17 (94.4)	1 (5.6)	18		36 (85.7)	6 (14.3)	42	
Pos	386 (89.4)	46 (10.6)	432		251 (75.4)	82 (24.6)	333	
Total	403 (89.6)	47 (10.4)	450		287 (76.5)	88 (23.5)	375	
P63				0.488				0.346
Neg	396 (89.6)	46 (10.4)	442		271 (77.0)	81 (23.0)	352	
Pos	5 (83.3)	1 (16.7)	6		15 (68.2)	7 (31.8)	22	
Total	401 (89.5)	47 (10.5)	448		286 (76.5)	88 (23.5)	374	
CK5/6				0.664				0.829
Neg	389 (89.6)	45 (10.4)	434		267 (76.3)	83 (23.7)	350	
Pos	13 (86.7)	2 (13.3)	15		18 (78.3)	5 (21.7)	23	
Total	402 (89.5)	47 (10.5)	449		285 (76.4)	88 (23.6)	373	
CK14				0.406				0.654
Neg	387 (89.8)	44 (10.2)	431		272 (76.4)	84 (23.6)	356	
Pos	14 (82.4)	3 (17.6)	17		13 (81.3)	3 (18.8)	16	
Total	401 (89.5)	47 (10.5)	448		285 (76.6)	87 (23.4)	372	
P-cadherin				**0.028**				0.641
Neg	378 (90.4)	40 (9.6)	418		223 (76.9)	67 (23.1)	290	
Pos	20 (76.9)	6 (23.1)	26		58 (74.7)	20 (25.6)	78	
Total	398 (89.6)	46 (10.4)	444		281 (76.4)	87 (23.6)	368	
CG				0.515				**0.003**
Neg	377 (90.0)	42 (10.0)	419		272 (77.9)	77 (22.1)	349	
Pos	24 (85.7)	4 (14.3)	28		11 (50.0)	11 (50.0)	22	
Total	401 (89.7)	46 (10.3)	447		283 (76.3)	88 (23.7)	371	
SYN				**0.009**				**0.030**
Neg	347 (91.1)	34 (8.9)	383		250 (78.4)	69 (21.6)	319	
Pos	54 (80.6)	13 (19.4)	67		35 (64.8)	19 (35.2)	54	
Total	403 (89.6)	47 (10.4)	450		285 (76.4)	88 (23.6)	373	
Sox2				**0.034**				0.313
Neg	159 (88.8)	20 (11.2)	179		119 (75.8)	38 (24.2)	157	
Pos	20 (74.1)	7 (25.9)	27		35 (68.6)	16 (31.4)	51	
Total	179 (86.9)	27 (13.1)	206		154 (74.0)	54 (26.0)	208	

Bold: statistically significant

Further analysis basing on the different Lum subtypes revealed that FGFR1 correlated with the high pN (*p* = 0.023), pT stages (*p* = 0.003), large tumor size (*p* = 0.005), the presence of LVI (*p* = 0.010), p-cadherin (*p* = 0.028), SYN (*p* = 0.009) and SOX2 (*p* = 0.034) expression in Lum A subtype only. There was no significant correlations with any clinicopathological features and most biomarkers (except for CG (*p* = 0.003) and SYN (*p* = 0.030)) in Lum B subtype (Table 3).

### Relationship of FGFR1 expression with patient outcome in different molecular breast cancer subtypes

**Figure 2:**
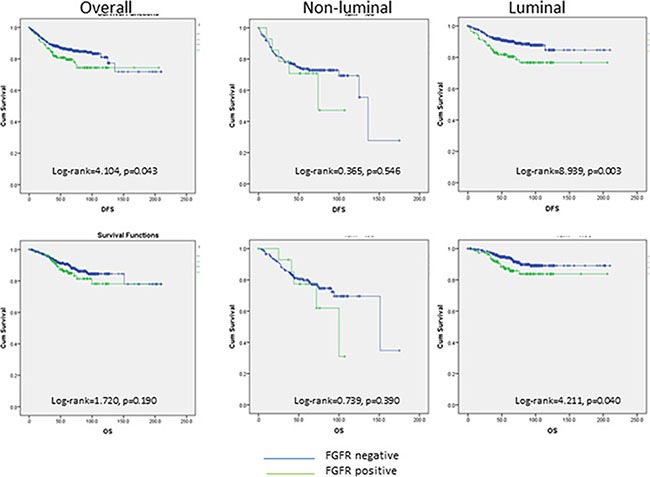
Kaplan-Meier analysis of DFS and OS in overall, non-liminal and luminal cancers

Follow-up data were available in 944 cases with a mean follow-up duration of 65.8 months (1–210 months). Overall, FGFR1 expression was associated with poor DFS (log- rank = 4.104, *p* = 0.043) but not OS (log- rank = 1.720, *p* = 0.190) (Figure 2). The associations with poor outcome were mainly observed in Lum cancers (DFS: log-rank = 8.939, *p* = 0.003; OS: log-rank = 4.211, *p* = 0.040) but not in non-Lum cancers (DFS: log-rank = 0.365, *p* = 0.546; OS: log-rank = 0.739, *p* = 0.390) (Figure 2).

**Figure 3:**
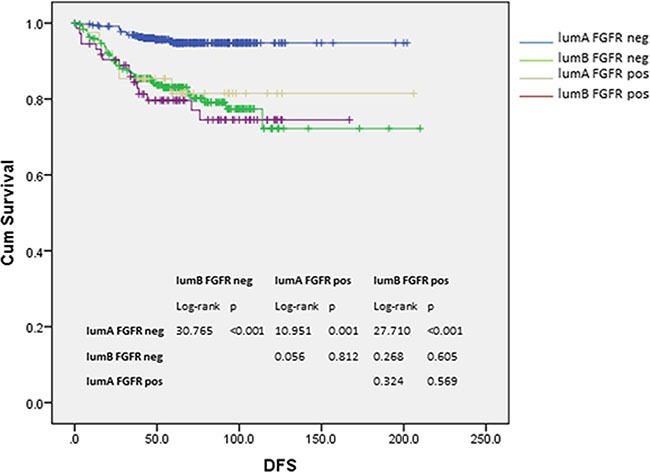
Kaplan-Meier analysis of DFS according to luminal subtypes and FGFR1 expression

In fact, when subtypes of Lum cancers were analyzed, the poor DFS (log-rank = 10.951, *p* = 0.001) in FGFR1-expressing cancers was only observed in Lum A cancers, but not in Lum B cancers with or without FGFR1 expression (log-rank = 0.268, *p* = 0.605). The worse DFS in FGFR1 expressing Lum A cancers was comparable to that of luminal B cancers (compared to FGFR1-expressing luminal B: log-rank = 0.324, *p* = 0.569; FGFR1 negative luminal B: log-rank = 0.056, *p* = 0.812) (Figure 3). Multivariate cox regression analysis on DFS also showed that FGFR1 expression in different luminal subtypes together with grade, pT and pN stages were independent prognostic factor in Lum cancers (Lum A FGFR1 neg as reference: Lum A FGFR1 pos: HR = 3.341, *p* = 0.008; Lum B FGFR1 neg: HR = 2.789, *p* = 0.001; Lum B FGFR1 pos: HR = 2.500, *p* = 0.013) (Table 4).

**Table 4:** Multivariate cox regression analysis for DFS in luminal cancers

	*p*-value	HR	95.0% CI	
			Lower	Upper
Initial step				
Grade	0.009	1.711	1.142	2.563
age	0.885	1.001	0.982	1.021
LVI	0.185	1.424	0.844	2.405
ER	0.232	0.659	0.332	1.306
PR	0.128	0.624	0.340	1.145
pT	< 0.001	2.140	1.418	3.229
pN	< 0.001	1.656	1.302	2.106
Lum A FGFR neg (ref)	0.026			
Lum B FGFR neg	0.014	2.270	1.182	4.358
Lum A FGFR pos	0.010	3.235	1.318	7.941
Lum B FGFR pos	0.022	2.410	1.132	5.131
Final step				
Grade	**0.006**	1.722	1.165	2.546
pT	**< 0.001**	2.207	1.474	3.304
pN	**< 0.001**	1.808	1.454	2.249
Lum A FGFR neg (ref)	**0.005**			
Lum B FGFR neg	**0.001**	2.789	1.538	5.058
Lum A FGFR pos	**0.008**	3.341	1.372	8.136
Lum B FGFR pos	**0.013**	2.500	1.209	5.173

## DISCUSSION

There are ongoing interests for FGFR as a prognostic marker and treatment target in breast cancer [18]. However, most studies focuses mainly on its gene amplification [3, 8–10, 12]. The FGFR amplicon is complex, composing of several candidate oncogenes which may drive cancer development [19]. In fact, while high FGFR1 protein expression was related to gene amplification, the reverse may not be true [10, 12, 20, 21]. Therefore, this study was designed to investigate FGFR1 protein expression in a large cohort of breast cancers by IHC staining. The relationship of FGFR1 expression with multiple relevant clinicopathologic features, tumor biomarker panels as well as the prognostic value in different molecular subtypes of breast cancer was investigated. The overall FGFR1 expression rate in breast cancer was 14.3%, occurring predominantly in Lum B cancers (24.9%). This observation was concordant with its reported gene amplification [10]. Little has been reported regarding the clinicopathologic and biomarker association of FGFR1 protein expression in breast cancer. One study that analyzed FGFR1 amplification by FISH on TMA did not demonstrate any association with histologic parameters, including grade, size, nodal status, vascular invasion or a number of biomarkers [3]. We observed significant correlation of FGFR1 expression with high tumor pN, pT stages, large tumor size, and increased expression of several biomarkers (ER, Ki67, P63, CG, SYN and SOX2). Its positive association with ER and Ki67 expression corroborated its prevalence in Lum B cancer subtype. FGFR signaling is one of the most common pathways implicated in controlling stemness [22]. Here, we observed a positive association of FGFR1 with the transcriptional factor SOX2 with neural stem cell renewal [23], and particularly with neuroendocrine differentiation in breast cancer.

Previously, we reported the specific association of SOX2 with expression of hormonal receptor and neuroendocrine differentiation in breast cancers [20]. Given the role of SOX2 in neural stem cell renewal, its expression have been reported in other types of neuroendocrine tumor [21–23]. Of interest, FGFR1 expression was also related to cancers with neuroendocrine differentiation. High copy number gain of FGFR1 was detected in pulmonary neuroendocrine tumors [24]. Ectopic expression of FGFR1 in mouse prostate cancer model was shown to associate with the acquisition of an aggressive neuroendocrine phenotype and metastasis [25]. However, the underlying mechanism for these observations was not completely clear. Notably, previous study has shown that blocking FGF signaling with FGFR1 inhibitor can reduce the level of SOX2 expression [26]. FGF signaling could control osteoblast differentiation through induction of SOX2 and regulation of the Wnt-β-catenin pathway [27]. Together with the current findings, we postulated that FGFR1 expression could regulate SOX2 expression and subsequently neuroendocrine differentiation in breast cancer.

Another interesting finding was the association of FGFR1 expression with poor outcome in Lum cancer. We found that FGFR1 expression was predominantly in luminal cancers, in particularly Lum B cancers. Concordantly, a significant association with the related biomarkers can be demonstrated. A significant association of FGFR1 with ER and Ki67 in the overall cohort while significant correlation with low PR and high Ki67 as well as a near significance with HER in luminal cancers were observed. Although there was a lack of association with PR and HER2 in the overall cohort, both luminal B and non-luminal cancers exhibited low PR and high HER2 expression. High FGFR1 was in the former and low was in the latter subtypes. The opposite relationship of FGFR1 with different subtypes could nullify its association with PR and HER2. By contrast, FGFR1 expression associated with Ki67 regardless of subtypes. FGFR1 activation has shown to induce proliferation in breast cancer [28]; thus its expression could have a direct cause-effect on increased Ki67 rather than merely an epiphenomenon. FGFR1 amplification was shown to be associated with poor outcome in hormone receptor-positive breast cancer and resistance to endocrine therapy [3, 9, 10]. Interestingly, here, we showed that its prognostic impact mainly associated with Lum A cancers. The FGFR1 expression in Lum A subtype was shown to be an independent prognostic feature. It had a similar hazard ratio as luminal B cancers for DFS. In addition, it correlated with poor prognostic features positively, including LVI, high pT, pN and P-cadherin expression mainly in Lum A. Lum B cancers are genetically and genomically altered to a greater extent than Lum A cancers [29]. Apart from FGFR1, other genes overexpressed in Lum B have also shown to affect cancer growth and patients outcome [30]. It appeared that multiple drivers could be involved in Lum B cancers. Lum A subtype is a diverse and the most frequent subtype in breast cancer. Within this subtype, four major subgroups, namely 1p/16q, copy number quiet, chr8-associated and copy number high (CNH), have been identified recently by genomic analysis [31]. CNH subgroup has shown to have poor prognosis. However, the prognostication in other subgroups, including Chr8-associated subgroup which associated with focal FGFR1 amplification, has not been reported. Our data showing poor outcome of FGFR1 expressing Lum A cancers may implicate the poor prognostication also for this Chr8-associated subgroup [31]. In the Chr8-associated subgroup, MAP3K1 mutation was frequently found. FGFR signaling can cause persistent MAPK activation, subsequently leading to tamoxifen resistance [4]. It could contribute to the poor outcome in the FGFR1 expressing Lum A cancers. Our results may be useful in further stratification and thus management of tamoxifen resistant Lum A cancers.

In summary, FGFR1 protein expression was shown to be associated with Lum cancers. Although it is more prevalent in Lum B subtype, its expression showed adverse prognostication significance in only Lum A cancers. FGFR-expressing Lum A cancers showed a similar outcome as Lum B cancers, suggesting its role in identifying the aggressive subset of the heterogeneous Lum A cancers. Agents targeting FGFR pathway are currently actively explored as breast cancer treatment, which could be especially relevant for tamoxifen resistant Lum A cancer.

## MATERIALS AND METHODS

### Patients and database

The histologic files of the 3 involved institutions were searched for breast carcinoma over a period of 4 (2002–2005), 7 (2003–2009), and 2 (2003–2004) years respectively. All consecutive cases with excision specimens were included. The study was approved by Joint Chinese University of Hong Kong—New Territories East Cluster clinical research ethics committee. All the specimens were routinely processed and stained with hematoxylin and eosin (H & E). All the slides form all the cases were reviewed, graded (modified Bloom and Richardson) [32], and histotyped (WHO 2012) by two pathologists separately in a blinded manner [33]. Lymphovascular invasion (LVI) and fibrotic focus (FF) were also evaluated as present or absent, as previously reported criteria [2]. Patients’ age, tumor size, lymph node involvement, pN stage, pT stage, and outcome data were retrieved from the medical records. Overall survival (OS) was defined as the time interval from the date of initial diagnosis to the date of breast cancer related death. Disease free survival (DFS) was defined as the duration from the date of initial diagnosis to the first detection of breast cancer specific relapse or death. If no relapse or death observed, the survival time was censored at the last follow up visit.

### Tissue microarray (TMA) construction and immunohistochemistry

TMAs containing representative tumor areas were constructed with duplicated 0.6-mm cores as previously described [18]. The TMAs were assembled with a tissue arrayer (Beecher Instruments, Silver Springs, MD). One section from each TMA was stained with H&E and reviewed to confirm the presence of representative tumors. Immunohistochemical (IHC) staining was performed on the TMA with the selected antibodies using Ultraview Universal DAB Detection Kit (Ventana, Tucson, AZ) after deparaffinization, rehydration, and antigen retrieval of the slides. All slides were counterstained with hematoxylin. The TMA slides were assessed for the staining intensity, and the actual percentage of stained cells in the nucleus, cytoplasm, or membrane according to different antibodies by 2 of the authors blinded to the clinical information and the staining results of other markers. For FGFR1 staining, the reactivity was assessed both membranous and cytoplasmic. The staining was considered positive when unequivocal staining was detected in at least 1% of tumor cells [13]. Several groups of other markers were examined, including basal markers (EGFR, c-kit, p63, CK5/6 and CK14), markers related to stem cell features (SOX2 and p-cadherin), neuroendocrine markers (chromogranin (CG) and synaptophysin (SYN)), hormonal receptors (ER and PR) and other common cancer markers (HER2 and Ki67). The staining was considered positive when there was moderate or strong immune reactivity at the appropriate location over the cut-off point. Any discordant results were resolved by reading the slides at a multi-head microscope and discussed. Further details of the IHC stainings and their assessment are shown in Supplementary Table S1.

In addition, all cases were also classified into molecular subtypes basing on IHC surrogates, listed as follows [34, 35].

Luminal A (Lum A) (ER+, PR ≥ 20%, HER2−, Ki67 < 20%),

Luminal B (Lum B) (ER+, PR < 20% and/or HER2+ and /or Ki67 ≥ 20%), HER2-overexpressed (HER2-OE) (ER−, PR−, HER2+),

Basal-like breast cancer (BLBC) (ER−, PR−, HER2−, CK5/6+, and EGFR+),

Unclassified (5NP) (ER−, PR, HER2−, CK5/6−, EGFR−).

### Statistical analysis

Statistical analysis was performed using SPSS for Windows, Version 21. For association between FGFR1 IHC staining and clinic-pathologic parameters, χ2 and Fisher exact tests were applied as appropriate. Survival analysis was accomplished using Kaplan–Meier method and comparison between groups was done using log-rank statistics. Multivariate cox regression analysis was performed to survival hazard ratios (HR) and corresponding 95% confidence intervals (95% CI) using the backwald method. Statistical significance was defined as *p* < 0.05.

## SUPPLEMENTARY MATERIALS TABLES


