# Supplementary material for: FGFR1 is an adverse outcome indicator for luminal A breast cancers
Source: Oncotarget. 2015 Dec 11;7(4):5063–73. doi: 10.18632/oncotarget.6563 (PMC4826266; doi:10.18632/oncotarget.6563)
Supplement: Supplementary file 1 [file oncotarget-07-5063-s001.pdf]

FGFR1 is an adverse outcome indicator for luminal A breast cancers

Supplementary Materials

Supplementary Table S1: Antibodies used for IHC analysis

Markers	Company	Clone	Dilution	Antigen retrieval	Incubation condition (min,°C	Assessment	Cutoff
ER	Neomarkers	SP1	Pre-diluted	EDTA pH8	32,37	N	1%
PR	Ventana	IE2	Pre-diluted	EDTA pH8	32,37	N	20%
Ki67	Ventana	41912	Pre-diluted	EDTA pH8	32,37	N	20%
EGFR	Ventana	3C6	Pre-diluted	EDTA pH8	32,37	M	5%
HER2	Ventana	4B5	Pre-diluted	EDTA pH8	16,37	M	3+
CK5/6	Dako	D5/16 B4	1:40	EDTA pH8	32,37	C,M	5%
CK14	Neomarkers	LL002	1:100	EDTA pH8	32,37	C,M	5%
c-kit	Dako	104D2	1:300	EDTA pH8	32,37	C,M	5%
P63	Ventana	4A4	Pre-diluted	EDTA pH8	32,37	N	5%
SYN	Novocastra	27G12	1:50	EDTA pH8	32,37	C,M	1%
CG	Biogene	MU-126-UC	1:200	EDTA pH8	32,37	C,M	1%
SOX2	Ventana	SP76	Pre-diluted	EDTA pH8	32,37	N	1%
p-cadherin	BD transduction lab	56/p-cad	1:200	EDTA pH8	32,37	C,M	10%
FGFR1	Ventana	D8E4	1:200	EDTA pH8	32, 37	C, M	1%

'N': nuclear; 'C': cytoplasmic; 'M': membraneous.

Supplementary Table S2: Association of FGFR1 expression of clinic-pathological features and biomarkers expression in luminal cancers

		FGFR1 (%)			
		Negative	Positive	Total	*p*-value
Clinico-pathological features					
Grade	1	135 (89.4)	16 (10.6)	151	**0.005**
	2	328 (84.8)	59 (15.2)	387	
	3	227 (79.4)	59 (20.6)	286	
FF	Absence	509 (85.1)	89 (14.9)	598	**0.086**
	Presence	165 (80.5)	40 (19.5)	205	
LVI	Absence	482 (85.8)	80 (14.2)	562	**0.031**
	Presence	174 (79.5)	45 (20.5)	219	
pN	0	364 (86.9)	55 (13.1)	419	**0.004**
	1	194 (81.5)	44 (18.5)	238	
	2	80 (84.2)	15 (15.8)	95	
	3	41 (70.7)	17 (29.3)	58	

pT	1	309 (87.5)	44 (12.5)	353	**0.001**
	2	332 (81.0)	78 (19.0)	410	
	3	27 (77.1)	8 (22.9)	35	
	4	6 (60.0)	4 (40.0)	10	
Molecular	Lum A	454 (89.0)	56 (11.0)	510	**< 0.001**
	Lum B	238 (75.1)	79 (24.9)	317	
Age	Mean	54.9	52.8	54.5	0.113
	SD	12.88	12.15	12.78	
	Range	22-97	28-91		
Tumor size	Mean	2.52	2.93	2.59	**0.001**
	SD	1.468	1.581	1.494	
	Range	0.2-13.9	0.3–9.5		
Biomarker					
ER	Neg	53 (88.3)	7 (11.7)	60	0.311
	Pos	639 (83.3)	128 (16.7)	767	
PR	< 20%	165 (77.1)	49 (22.9)	214	0.003
	≥ 20%	523 (85.8)	86 (14.2)	609	
HER2	Neg	611 (84.6)	111 (15.4)	722	0.053
	Pos	81 (77.1)	24 (22.9)	105	
Ki67	< 20%	547 (87.3)	82 (12.7)	629	**< 0.001**
	≥ 20%	140 (72.9)	52 (27.1)	192	
c-kit	Neg	595 (83.7)	116 (16.3)	711	0.739
	Pos	89 (82.4)	19(17.6)	108	
P63	Neg	668 (84.0)	127 (16.0)	795	0.077
	Pos	20(71.4)	8 (28.6)	28	
CK5/6	Neg	658 (83.7)	128 (16.3)	786	0.729
	Pos	31 (81.6)	7 (18.4)	38	
CK14	Neg	661 (83.8)	128 (16.2)	789	0.766
	Pos	27 (81.8)	6 (18.2)	33	
P-cadherin	Neg	602 (84.9)	107 (15.1)	709	**0.011**
	Pos	78 (75.0)	26 (25.0)	104	
CG	Neg	651 (84.5)	119 (15.5)	770	**0.007**
	Pos	35 (70.0)	15 (30.0)	50	
SYN	Neg	601 (85.4)	103 (14.6)	704	**0.001**
	Pos	89 (73.6)	32 (26.4)	121	
Sox2	Neg	280 (82.8)	58 (17.2)	338	**0.013**
	Pos	55 (70.5)	23 (29.5)	78	
